# Immunocytochemical and Ultrastructural Evidence Supporting That Andes Hantavirus (ANDV) Is Transmitted Person-to-Person Through the Respiratory and/or Salivary Pathways

**DOI:** 10.3389/fmicb.2019.02992

**Published:** 2020-01-10

**Authors:** Enrique Pizarro, Maritza Navarrete, Carolina Mendez, Luis Zaror, Carlos Mansilla, Mauricio Tapia, Cristian Carrasco, Paula Salazar, Roberto Murua, Paula Padula, Carola Otth, Esteban Martin Rodríguez

**Affiliations:** ^1^Instituto de Anatomía, Histología y Patología, Facultad de Medicina, Universidad Austral de Chile, Valdivia, Chile; ^2^Instituto de Microbiología Clínica, Facultad de Medicina, Universidad Austral de Chile, Valdivia, Chile; ^3^Unidad Microbiología Clínica, Hospital Base Valdivia, Servicio de Salud Valdivia, Valdivia, Chile; ^4^Unidad de Anatomía Patológica, Servicio de Salud Aysén, Hospital Regional de Coyhaique, Aysén, Chile; ^5^Subdepartamento Anatomía Patológica Hospital Base Valdivia Servicio de Salud Valdivia, Valdivia, Chile; ^6^Instituto de Ecología y Evolución, Facultad de Ciencias, Universidad Austral de Chile, Valdivia, Chile; ^7^Servicio Biología Molecular, Departamento de Virología, Instituto Nacional de Enfermedades Infecciosas, ANLIS “Dr. Carlos G. Malbrán”, Buenos Aires, Argentina

**Keywords:** hantavirus, ANDV, person-to-person transmission, alveolar epithelium, macrophages, salivary glands

## Abstract

In South America Andes hantavirus (ANDV) is hosted by the rodent *Oligoryzomys longicaudatus* (also known as pygmy rice rat). In humans, ANDV causes Hantavirus Pulmonary Syndrome (HPS), with a fatality rate of about 40%. Epidemiologic and molecular evidence has shown that ANDV can be transmitted from person to person. Sin Nombre hantavirus, occurring in North America, and ANDV are genetically related, and both cause HPS with similar clinical evolution and mortality rate. However, only ANDV is transmitted from person to person. A recent hantavirus outbreak in a small village in Southern Argentine, with 29 HPS cases and 11 deaths has brought to mind that person-to-person transmission continues to be a public health emergency. The present investigation was aimed to understand how does ANDV actually spread between persons. Tissue samples of lung and salivary glands from infected *Oligoryzomys longicaudatus* and lethal cases of human HPS were investigated by bright field immunocytochemistry, multichannel immunofluorescence, and transmission electron microscopy. The findings are consistent with ANDV infection and replication in the lung alveolar epithelium and macrophages, and in the secretory cells of the submandibular salivary glands. In the lung of infected *Oligoryzomys longicaudatus* and human cases HPS, the bulk of immunoreactive hantavirus antigens was localized in epithelial cells of the alveolar walls and macrophages. The ultrastructural study supports that in the lung of HPS patients the virus replicates in the alveolar epithelial cells with virus particles being discharged into the alveolar lumen. Virus-like particles were seen within vacuoles of the lung macrophages. Considering that these macrophages can reach the conductive segments of the airways, their expectoration becomes a deadly bullet for ANDV transmission. In the submandibular glands of infected rodents and HPS cases, ANDV antigens were in capillary endothelium, the secretory cells and filling the lumen of the excretory pathway. It is proposed that in patients with HPS caused by ANDV the alveolar epithelium and macrophages would be the gate for the airway spreading of the virus, while the salivary glands are a target for virus replication and an exit pathway through saliva.

## Introduction

Lee et al. (1978) isolated from the lung of 73 mice (*Apodemus agrarius coreae*) the causative agent of the Korean hemorrhagic fever. The virus found was named Hantaan virus (HTNV), after the river close to the area where the mice were captured.

Shortly after this finding, a human disease primarily caused by HTNV, the hemorrhagic fever with renal syndrome (HFRS), was found to occur in Europe (Sundström, 2013; Vaheri et al., 2013a,b; McAllister and Jonsson, 2014). More than 10,000 HFRS cases are reported annually in Europe (Vaheri et al., 2013a).

In 1993, an outbreak of a disease with severe respiratory distress of unknown origin emerged in the Four Corners Region (Arizona, Colorado, New Mexico, and Utah) of the United States (Schmaljohn and Hjelle, 1997). Half of the patients died within 2 days after onset of symptoms (Khan et al., 1996). The molecular biology tools allowed a rapid identification of the causative agent, a novel hantavirus (Nichol et al., 1993; Nichol, 2000). The virus was called Sin Nombre virus (SNV), and the disease was named Hantavirus pulmonary syndrome (HPS) (Schmaljohn and Hjelle, 1997; McAllister and Jonsson, 2014; Avsic-Zupanc et al., 2019).

In 1995, a fatal case of HPS was reported in southwestern Argentina. The nucleotide sequence revealed a novel hantavirus, referred as Andes virus (ANDV) (López et al., 1996). Outbreaks of HPS caused by ANDV occurred in Argentina (1996) and Chile (1997) (Wells et al., 1997; Toro et al., 1998). The rate mortality was 50%.

Hantaviruses appear to have co-evolved with the rodent reservoir host species over 20 million years (Schmaljohn and Hjelle, 1997; Nichol et al., 2000). In the natural rodent reservoir, hantavirus infection is persistent and asymptomatic. At variance, infected humans, being incidental transient hosts, developed a severe disease. Each hantavirus species is associated with a specific rodent reservoir. In Southern United States, SNV was isolated from deer mice (Nichol et al., 1993; Jonsson et al., 2010). In Argentina and Chile, ANDV is harbored by the rodent *Oligoryzomys longicaudatus* (Levis et al., 1998; Padula et al., 2000).

Exposure to aerosols carrying hantavirus is believed to be the primary route of transmission from hantavirus-infected rodents to humans (Armstrong et al., 1995).

Despite extensive epidemiologic studies of hantaviruses occurring in Europe and America, person-to-person transmission of hantaviruses had been considered unlikely until 1996. However, in an outbreak occurring in Southern Argentina in 1996, and reported in 1997, the epidemiologic evidence strongly suggested person-to-person transmission of ANDV (Wells et al., 1997). Case-fatality rate was 50%. This was the first recognition that these viruses may cause person-to-person transmission of the illness. Direct genetic evidence of person-to-person transmission of ANDV was soon obtained (Padula et al., 1998). An outbreak of 25 cases of HPS that occurred in Southern Chile confirmed person-to-person transmission of ANDV (Toro et al., 1998). New clusters with person-to-person transmission were later reported (Martínez et al., 2005).

Epidemiologic and genetic evidence indicates that person-to-person spread of ANDV takes place during the prodromal phase of the disease (Martínez et al., 2005). For person-to-person transmission to occur, close contact is required. Indeed, the risk of infection among household contacts of index case patients with HPS is increased in sex partners, particularly in those who engaged in deep kissing (Ferrés et al., 2007; Jonsson et al., 2010).

The incidence in Southern South America of HPS caused by ANDV has kept constant throughout the years, with a modest decreased in rate mortality (30–40%). Despite person-to-person transmission of ANDV is known since 1997, this way of infection continues operating today mainly because the transmission would occur during the incubation period, when the infected patient has not yet developed clinical symptoms.

Although person-to-person transmission of ANDV was demonstrated 22 years ago, the actual mechanism of transmission between humans continues to be largely ignored. SNV and ANDV are genetically related, and both cause an HPS with similar clinical evolution and mortality rate. However, only ANDV is transmitted from person to person. How to explain this fundamental epidemiological difference? Would both hantaviruses have a different cell tropism so that the ANDV-infected cells would facilitate person-to-person transmission? Considering that the epidemiological data discussed above point to respiratory droplets and saliva as potential ways of ANDV human transmission, the tracking of ANDV proteins in the cells of lung and salivary glands of fatal HPS cases by using immunocytochemical tools appeared as a promising task.

In 2004 we published a paper on transmission of ANDV in *Oligoryzomys longicaudatus* reservoir populations and reported on some evidence indicating the presence of the virus in the alveolar epithelium and in salivary glands (Padula et al., 2004). In 2007, at an International Conference on HFRS, HPS and Hantaviruses, we presented a poster reporting on immunocytochemical evidence on the presence of ANDV in alveolar epithelium and in salivary glands of fatal HPS cases (Navarrete et al., 2007). Surprisingly, these two preliminary reports have remained the only immunocytochemical evidence for the person-to-person transmission of ANDV. The hantavirus outbreak that occurred in Epuyén, in the Andean region of Southern Argentine, between October 2018 and January 2019, with 29 confirmed cases and 11 deaths^1^ has reinforced the hypothesis of person-to-person transmission. This public health emergency stimulated us to revisit our collection of tissue samples collected from infected *Oligoryzomys longicaudatus* mice and lethal cases of human HPS, and studied them by bright field immunocytochemistry and multichannel immunofluorescence for ANDV antigens and other proteins, and with transmission electron microscopy hoping to further clarify the ANDV pathogenesis and the transmission mechanisms. Consistent evidence was obtained that (i) ANDV infection and replication would take place in the alveolar epithelium with virus-like particles reaching the alveolar lumen, (ii) macrophages would also be a site of ANDV infection and replication, and carry virus-like particles inside cytoplasmic large vacuoles, and (iii) ANDV concentrate and probably replicate in secretory cells of the submandibular salivary gland and reach the lumen of the gland excretory pathway. This evidence supports the view that ANDV is transmitted through respiratory droplets and saliva aerosols.

## Materials and Methods

### Human and Animal Samples

The human and animal samples were obtained from 1999 to 2004. The experimental protocol was approved by the Ethic Committee and the Animal Care and Experimentation Committee of Universidad Austral de Chile (ref.: FONDEF D 99I 1105, 1999-2002). The guidelines of the Chilean National Research Council (Conicyt) for the handling of animal and biosafety were followed. All procedures were conducted in agreement with the laboratory animals limited use recommendations^2^.

### Tissue Samples From HPS Patients

Tissue samples were obtained from 10 lethal HPS cases from southern of Chile. Viral infection was confirmed by capture-IgM ELISA for Andes-specific IgM antibodies. The HPS cases were three female and seven male, with an average age of 36 years. Samples from the lung were obtained from ten patients, with a post mortem interval ranging from 24 to 48 h. In two of these cases samples of the submandibular salivary gland were also obtained. Some tissue samples were fixed by immersion in Bouin fixative for 2–3 days; others were first fixed in formalin and then postfixed in Bouin fixative for 2–3 days. Samples were dehydrated in a series of alcohols and embedded in paraffin. Serial sections (2 μm or 6 μm thick) obtained from each tissue block were mounted on silanized (3-aminopropyltriethoxysilane; Polysciences Inc., Warrington, PA, United States) slides. Adjacent sections were used for histological and immunocytochemical staining. In two out of the 10 lethal HPS cases processed for light microscopy, lung samples for electron microscopy were also obtained.

### Collection and Serological Screening of Wild *Oligoryzomys longicaudatus* Samples

Using Sherman traps located in various localities of Los Rios and Los Lagos Regions of Chile, *Oligoryzomys longicaudatus* mice were collected. Rodents were tested for seropositivity collecting 70–150 μl blood samples by retro-orbital puncture under ether anesthesia (for more details see Padula et al., 2004). The presence of antibodies against hantavirus antigens was detected by using an ANDV specific ELISA test as described before (Padula et al., 2000). Recombinant ANDV nucleoprotein (N-ANDV) was used as specific antigen. A peroxidase-labeled antibody against *Peromyscus leucopus* IgG was used to detect mouse IgG. ABTS (2.2’-azino-di [3-ethyl-benzthiazoline sulfonate]) was used as substrate for peroxidase; absorbance was measured at 405 nm.

### *Oligoryzomys longicaudatus* Tissue Sampling

Five non-infected and eight infected mice were used. Six *Oligoryzomys longicaudatus* had been infected in nature and two were experimentally infected (Padula et al., 2004). Briefly, one sero-positive rodent infected in nature and one sero-negative rodents were caged together in the drum for 24 h. After 35 days, once the experimentally infected *Oligoryzomys longicaudatus* displayed hantavirus IgG antibodies, was sacrificed and processed as described below. This protocol was done twice. All animals were anesthetized prior to sacrifice. Infected and non-infected rodents were fixed by vascular perfusion with Bouin fixative for 30 min; then the tissue samples were dissected out and immersed in Bouin fixative for 2–3 days. Samples from lung, submandibular, sublingual and parotid salivary glands were obtained. All samples were dehydrated in a series of alcohols and embedded in paraffin. Serial sections from each tissue blocks (2 μm of 6 μm thick) were obtained. Adjacent sections mounted on separate silanized (3-aminopropyltriethoxysilane; Polysciences Inc., Warrington, PA, United States) slides were used for histological staining and immunocytochemistry.

### Immunohistochemical Procedures

Three methods were used, (i) the immunoperoxidase method of Sternberger et al. (1970); (ii) sequential staining of the same section using different antibodies in each sequence; (III) double immunofluorescence.

### Immunoperoxidase Staining

Masked antigens were exposed using two protocols. (i) Enzymatic digestion. Sections were digested in 10 μg/ml proteinase K (Dako, Copenhagen, Denmark) in Tris, pH 7.5, 30 min (Zaki et al., 1995), (ii) Microwave irradiation. The slides were immersed in a coplin jar filled with Tris-HCl buffer, pH 9.5, containing 5% urea and irradiated in a micro-wave oven (900 W) for two sessions, 5 min each. Endogenous peroxidase was blocked by exposing the sections to 10% H_2_O_2_ in methanol.

The biotin-streptavidin-peroxidase method was used (DAKO Kit K0679). Three primary antibodies were used: (i) rabbit antiserum against recombinant N-ANDV (anti-N-ANDV, Padula et al., 2004), 1:1000 dilution; (ii) rabbit antiserum against a cell-cultured ANDV (anti-ANDV, Padula et al., 2004), 1:1000 dilution; (iii) goat antiserum against Laguna Negra virus nucleoprotein (anti-N-LNV), gently provided by Center for Disease Control and Prevention, Atlanta, GA, United States), 1:1000 dilution. Incubation in the primary antibodies was in a moist chamber, for 18 h, at room temperature. Antibodies were diluted in Tris buffer, pH 7.8, containing 0.7% non-gelling seaweed gelatine lambda carrageenan (Sigma, St. Louis, MO, United States) and 0.5% Triton X-100 (Sigma, St. Louis, MO, United States). Secondary antibodies were rabbit anti-goat and goat anti-rabbit immunoglobulins conjugated with Biotin (Dako, Copenhagen, Denmark). The secondary antibody was detected with streptavidin-HRP conjugate and visualized by using hydrogen peroxide as substrate (Merck, Darmstadt-Germany) and 3-amino-9-ethilcarbazol (reddish reaction product) as electron donor. Control tests were: (i) sections of the lung and salivary glands of infected *Oligoryzomys longicaudatus* and HPS human cases were processed as for immunocytochemistry but incubation in the primary antibody was omitted; (ii) sections of the lung and salivary glands of non-infected rodents were processed for the complete immunocytochemistry procedure.

The general organization of the tissue was visualized by staining the immunostained sections with Toluidine blue, hematoxylin of Nomarski optic.

### Sequential Immunostaining of the Same Section

Sections of mouse salivary gland were incubated in one of the primary antibodies indicated below, for 3 h at room temperature. After washing, sections were incubated with swine FITC-labeled anti-rabbit IgG (Dako, Copenhagen, Denmark), for 30 min and visualized under a fluorescence microscope. A series of pictures, at different magnifications, were taken. Then sections were washed in phosphate buffered saline and the primary and secondary antibodies were eluted with Gomori’s oxidizing mixture (Rodríguez et al., 1984). After repeated washes, sections were sequentially incubated in a different primary antibody, a biotin-conjugated anti-rabbit IgG and streptavidin-HRP conjugate (Dako, Copenhagen, Denmark). 3-amino-9-ethilcarbazol was used as electron donor. Pictures of the same selected areas photographed under the fluorescence microscope were now taken using bright field microscopy. In some sections, effective elution of antibodies used in the first sequence was tested in two ways: (i) by analysis under fluorescence microscopy of sections treated with the Gomori’s mixture; (ii) by incubation of these treated sections with FITC labeled anti-rabbit IgG for 30 min followed by analysis under fluorescence microscopy. In both cases, lack of fluorescence signal was regarded as an efficient elution of antibodies used in the first sequence. The pairs of sequential sera used were (i) anti-ANDV → anti-N-LNV; (ii) anti-ANDV → anti-N-ANDV; (iii) anti-N-LNV → anti-ANDV.

### Staining With Silver Methenamine Followed by Immunostaining

With the aim to visualize the frontier between blood vessels and epithelial cells of the alveolar walls, sections were stained with silver methenamine after oxidation with periodic acid (Rodríguez et al., 1984); this method selectively stains the basal lamina of the epithelial/endothelial cells. This procedure was followed by immunostaining applying the immunoperoxidase methods as described above, using anti-N-ANDV.

### Double Immunofluorescence

Sections were incubated overnight at room temperature with primary antibodies (raised in mice or rabbits) for 18 h. The following pairs of antibodies were used: (1) mouse monoclonal anti-amylase (amylase G-8: Sc-166349, recognizing precursor and mature forms of salivary amylase) (Santa Cruz Biotechnology, Dallas, TX, United States), 1:500 dilution/anti-N-ANDV, 1:1000 dilution; (2) CD68, a monoclonal antibody specific human monocytes and macrophages (Dako, Copenhagen, Denmark) (Micklem et al., 1989), 1:100 dilution/anti-ANDV, 1:1000 dilution; (3) anti-N-ANDV, 1:1000 dilution/β3 integrin, CD61 monoclonal antibody (Invitrogen, Carlsbad, CA, United States), 1:100 dilution. After washes in Tris buffer, pH 7.8, sections were incubated with anti-mouse IgG and anti-rabbit IgG conjugated with Alexa Fluor 594 and 488, respectively (1:500 dilution) (Invitrogen, Carlsbad, CA, United States). Slides were studied under an epifluorescence microscope using the multidimensional acquisition software AxioVision Rel of Zeiss (Aalen, Germany). For better background information, a phase contrast image was obtained using a third channel so that the images acquired with both Alexa Fluor could be merged with the phase contrast picture.

### Transmission Electron Microscopy

Several tissue blocks about 2 mm/side were obtained about 4 h after death from the lung of a fatal case of HPS and fixed by immersion in 4% paraformaldehyde and 2.5% glutaraldehyde buffered to pH 7.4 with 0.1 M monosodium-disodium phosphate, for 2 h, at room temperature. In a second fatal HPS case, lung samples that had been kept overnight in buffered formalin were trimmed down into 2 mm/side block and further fixed in in 4% paraformaldehyde and 2.5% glutaraldehyde for 2 h. In both cases, post-fixation was in 1% OsO4 dissolved in 0.1 M phosphate buffer, for 2 h, at 4°C. Embedding was in a mixture of Epon and Araldite. Ultrathin sections were contrasted with uranyl acetate and lead citrate.

## Results

### Localization of Hantavirus Antigens in the Lung of ANDV Infected

#### Oligoryzomys longicaudatus

**FIGURE 1:**
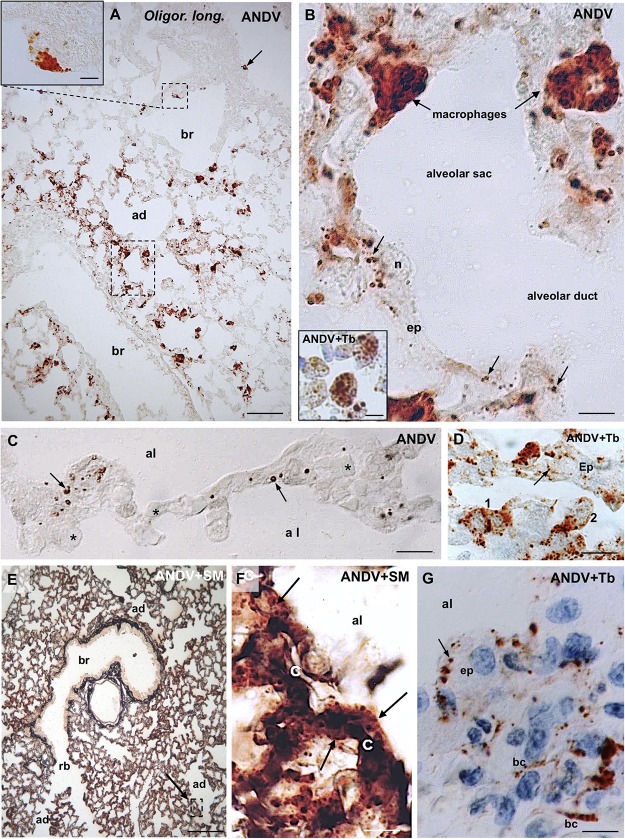
Lung samples from infected *Oligoryzomys longicaudatus.* Paraffin sections of rodents fixed by vascular perfusion with Bouin fluid. Immunostaining with anti-ANDV. **(A)** Low magnification view of a 7 μm thick section showing the uneven distribution of ANDV + material throughout the lung. Macrophages loaded with immunoreactive granules are seen in the lung parenchyma and attached to the luminal wall of bronchioles (arrow, small square). Area framed by rectangle is shown in **(B)**. *Inset.* Detailed magnification of area framed in **(A)**. **(B)** High power view of area framed in **(B)**. Macrophages containing ANDV + masses and granules are seen in the alveolar septa and traversing the alveolar wall. The epithelial cells (ep) lining the alveolar wall contain ANDV + granules of varying size (arrows). *Inset.* Immunoreactive macrophages are seen in the lumen of alveolar sacs and ducts. **(C)** 2 μm thick section immunostained for ANDV and visualized with Nomarski optic. ANDV + granules are seen in the cells lining the alveolar walls (arrows). Asterisks indicate blood capillaries. **(D)** 2 μm thick section immunostained for ANDV and background staining with Toluidine Blue. 1 and 2 point to immunoreactive macrophages at the alveolar wall and reaching the alveolar lumen, respectively. Arrow points the ANDV material in an epithelial cell. **(E)** Combined use of silver methenamine staining and ANDV immunostaining. Low magnification view of a 7 μm thick section showing the well-preserved lung histology. An area similar to that framed is shown in **(F)**. **(F)** Basement membranes of the alveolar and capillary (c) walls are stained in black by silver methenamine. Arrows point to ANDV + granules in cells located between basement membrane and alveolar lumen. **(G)** 7 μm thick section showing the ANDV + material in alveolar epithelial cells (ep. arrow) and in blood capillaries (bc). ad, alveolar duct; al, alveolar lumen; br, bronchiole; rb, respiratory bronchiole; Tb, toluidine blue; SM, silver methenamine. *Scale bars.* **(A)** 70 μm; inset 8 μm; **(B)** 7 μm; inset 7 μm; **(C)** 15 μm; **(D)** 14 μm; **(E)** 170 μm; **(F)** 12 μm, **(G)** 8 μm.

Description of results obtained in infected rodents will be mainly based on those specimens fixed by vascular perfusion. Immunoreactive hantavirus antigens were unevenly distributed throughout the lung, with areas being strongly reactive hantavirus antigens alternating with others devoid of immunoreaction (Figure 1A). No antigens were detected in the cells lining the airway, namely, bronchi and bronchioles (Figure 1A).

The bulk of immunoreactive hantavirus antigens was localized in epithelial cells of the alveolar walls and in macrophages (Figures 1A,B). With anti-ANDV the immunoreaction in the epithelial cells appeared as fine dots 0.6–1.5 μm in diameter (Figures 1B,C,G).

Lung macrophages loaded with viral immunoreactive material were readily visualized; they were seen in the alveolar septa (Figures 1B,D), traversing the alveolar wall (Figures 1B,D), in the alveolar lumen (Figure 1B, inset) or attached to the luminal surface of bronchioles and bronchi (Figure 1A). Within macrophages viral antigens appeared as granules 0.8–1.6 μm (Figure 1A, inset), or as masses about 2–3 μm in diameter (Figure 1B and inset).

In 2 μm-thick sections immunostained for ANDV immunoreactive macrophages and alveolar epithelial cells were readily distinguishable (Figure 1D).

Immunoreactive antigens within endothelial cells of lung blood vessels were occasionally seen (Figure 1G).

In sections first stained with silver methenamine and then immunostained with anti-ANDV, the basal lamina separating capillaries from cells lining the alveolar lumen appeared distinctly black stained. Cells located between the basal lamina and the alveolar lumen, corresponding to epithelial cells, contained abundant immunoreactive hantavirus antigens (Figures 1E,F).

No immunoreactive material with any of the antibodies used was seen in tissue samples of non-infected *Oligoryzomys longicaudatus*.

The cell distribution and amount of immunoreactive ANDV proteins in the lung and salivary gland of the naturally infected *Oligoryzomys longicaudatus* and that of the experimentally infected rodents were similar.

### Localization of Hantavirus Antigens in the Lung of HPS Cases

**FIGURE 2:**
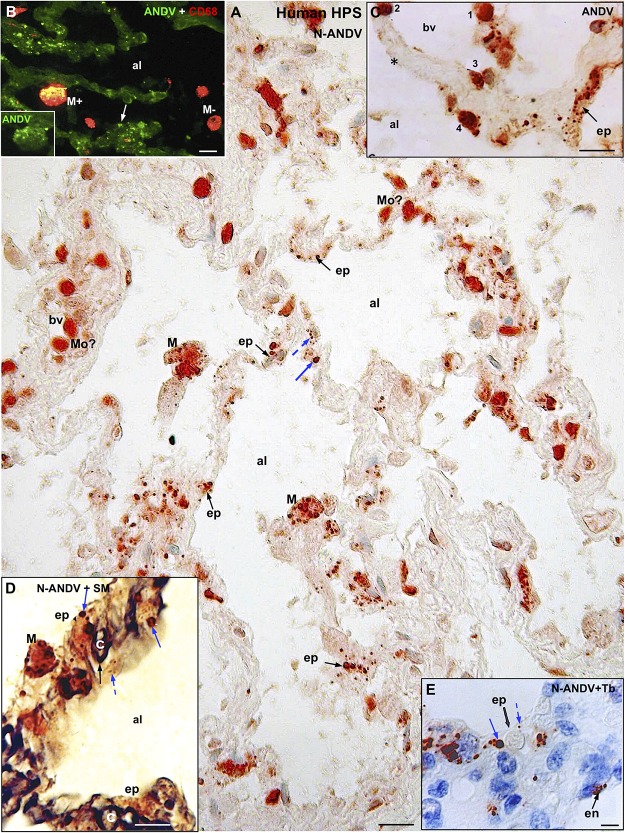
Human lung from lethal Hantavirus pulmonary syndrome (HPS) cases. **(A)** Paraffin section immunostained with anti-ANDV. Immunoreactive Hantavirus antigens are present in epithelial cells (ep) lining the alveoli, septal and luminal macrophages (M) and in cells located within blood vessels (bv) that might correspond to monocytes (Mo?). Broken and full blue arrows point to small and large immunoreactive inclusions in epithelial cells, respectively. **(B)** Double immunofluorescence for ANDV (green) and CF68 (red). Particulate ANDV antigens are seen in alveolar septa (arrow). There are luminal macrophages that contain virus antigens (M+) and others that do not (M–). *Inset.* M+ macrophage visualized only with the channel for ANDV. **(C)** A large blood vessel (bv) located close to an alveolar septum is shown. The alveolar epithelium (ep) contains viral granules (arrow). Cells 1 to 4 likely correspond to circulating ANDV + monocytes at different stages of migration into the alveolar lumen (al). **(D)** 7 μm thick paraffin section stained first with silver methenamine (SM) and then immunostained with anti-N-ANDV. The basal lamina of the epithelial and endothelial cells appears black (full black arrow). Cell located between the basal lamina and the alveolar lumen (al) contains masses (full blue arrow) and granules (broken blue arrow) of immunoreactive Hantavirus antigens (broken arrows). **(E)** 7 μm thick paraffin section immunostained with anti-N-ANDV and background stained with Toluidine blue (Tb). Nomarski optic. Viral antigens are seen in alveolar epithelial cells (ep) and in endothelial cells (en). Broken and full blue arrows point to small and large immunoreactive inclusions in epithelial cells, respectively. al, alveolar lumen; bv, blood vessel; ep, alveolar epithelium; en, endothelium; Mo, monocyte?; M, macrophage; Tb, toluidine blue; SM, silver methenamine. *Scale bars.* **(A)** 20 μm; **(B)** 14 μm; **(C)** 16 μm; **(D)** 10 μm; **(E)** 6 μm.

In sections first stained with silver methenamine and then immunostained with anti-ANDV or anti-N-ANDV, the basal lamina separating capillaries from cells lining the alveolar lumen appeared distinctly black stained. These cells, corresponding to epithelial cells, contained abundant immunoreactive hantavirus antigens (Figure 2D).

Hantavirus antigens were mainly localized in epithelial cells of alveoli and in macrophages (Figure 2A). Occasionally, antigens particles were seen in endothelial cells (Figure 2E). With anti-ANDV the immunoreactive material in epithelial cells appeared as granules 0.7–1.5 μm size (Figures 2A–C). When sections were immunostained with anti-N-ANDV the epithelial cells displayed 0.7-1.5 μm inclusions and, in addition, masses of immunoreactive antigens ranging in diameter between 2 and 3 μm (Figures 2A,D,E).

**FIGURE 3:**
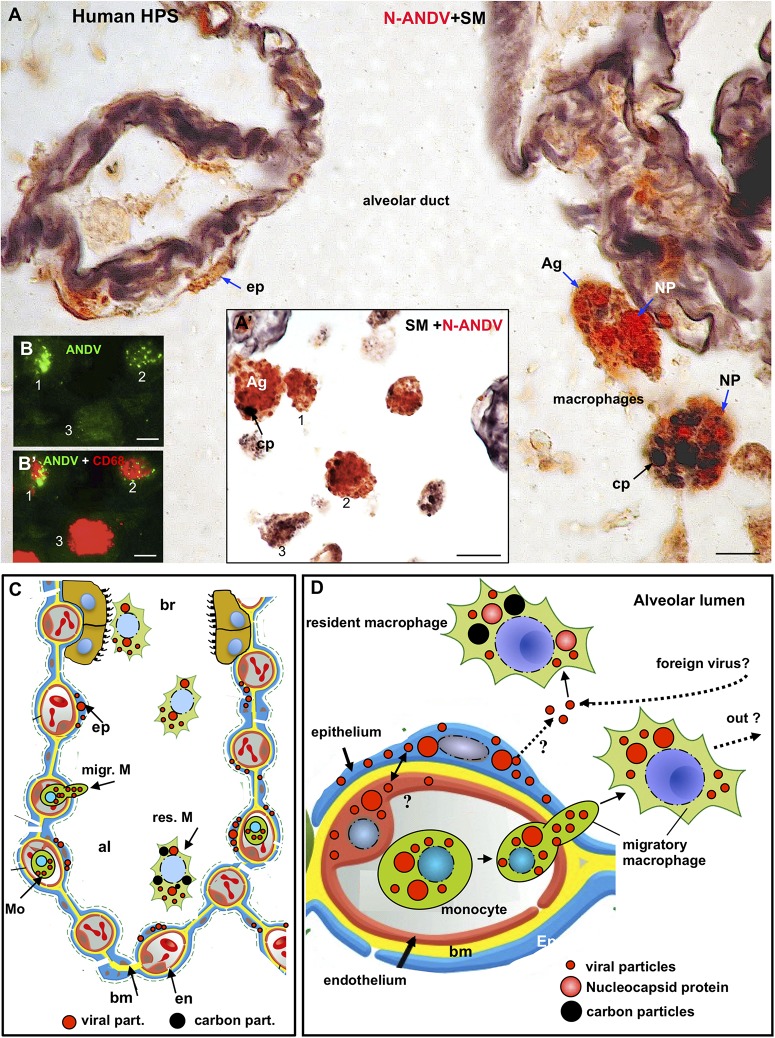
Human lung from lethal Hantavirus pulmonary syndrome (HPS) cases. **(A)** 7 μm thick paraffin section stained with silver methenamine (SM) and then immunostained with anti-N-ANDV. Detailed magnification of an alveolar duct showing viral material in epithelial cells (ep) and in luminal macrophages. The latter are loaded with antigen granules (Ag), N-ANDV + masses (NP) and carbon inclusions (cp) of varying size. **(A’)** Luminal macrophages display a varying degree of N-ANDV antigens (Ag) and carbon particles (cp) load (1–3). **(B,B’)** Double immunofluorescence for ANDV (green) and CF68 (red) of luminal macrophages. There are CD68+, ANDV + macrophages (1, 2) and CD68+ cells devoid of viral granules (3). *Scale bars.* **(A)** 10 μm; **(A’)** 20 μm; **(B,B’)** 10 μm. **(C,D)** Schematic representation of the immunocytochemical tracking of virus antigens in the lung of patients with Hantavirus pulmonary syndrome (HPS). **(C)** The walls of an alveolus (al) and a bronchiole (br) are represented. Red, endothelium, yellow basement membrane, blue alveolar epithelium, light brown, ciliated epithelium. Viral antigens are shown in epithelium (ep), circulatory monocytes (Mo), migratory macrophages (migr. M) and macrophages in the respiratory pathway (br). Resident microphages are shown to contain viral (red) and carbon (black) particles. **(D)** Detailed drawing showing viral-like particles (small red dots), viral masses (NP, nucleocapsid protein?) (large red dots) in endothelium, epithelium, monocytes and migratory macrophages. Resident macrophages containing carbon particles (black dots) also contain virus antigens. The possibility that resident macrophages incorporate those Hantaviruses that have reached the alveolar lumen either from the epithelial cells or from the airway, is represented. The large masses of virus antigens, likely corresponding to nucleocapsid protein would suggest viral replication in endothelium, epithelium and macrophages (see also Figure 5B).

CD68 immunoreactive macrophages were seen in the wall of alveoli and in the alveolar lumen (Figures 2B, 3B’). About 70% of CD68 + macrophages contain hantavirus antigens (Figures 2B, 3B,B’). With anti-ANDV the immunoreactive material in macrophages appeared as fine granules 1-2 μm in size (Figures 2B, 3B), while anti-N-ANDV revealed, in addition, distinct masses about 3-5μm in diameter (Figures 2A, 3A,A’).

The size of macrophages varied from 10 to 25 μm, regardless their location (Figures 3A’,B,B’). In the lumen and wall of blood vessels there were spherical cells about 10-12μm in diameter that reacted strongly with anti-ANDV and anti-N-ANDV (Figures 2A,C). Similar cells were seen attached to the blood vessel wall, in the alveolar septa and attached the alveolar wall (Figures 2A,C).

A relevant finding for the purpose of the present investigation was the presence in the alveolar lumen of large macrophages (about 25 μmin size) that were loaded with (1) black carbon particles 1 to 5 μm in size, and (2) small and large masses of hantavirus antigens (Figures 3A,A’,C,D). The relative load of carbon and viral antigen varied from cell to cell (Figures 3A,A’). As judged from the 7 μm thick sections there were a few alveolar macrophages that contained virus antigen but not carbon particles and others the contained carbon but not virus antigens (Figures 3A,A’). No serial sections through the same cells were performed to confirm this observation.

No macrophages with antigen material and carbon particles were seen in the lung parenchyma.

### Transmission Electron Microscopy of the Lung of Fatal Case of HPS

**FIGURE 4:**
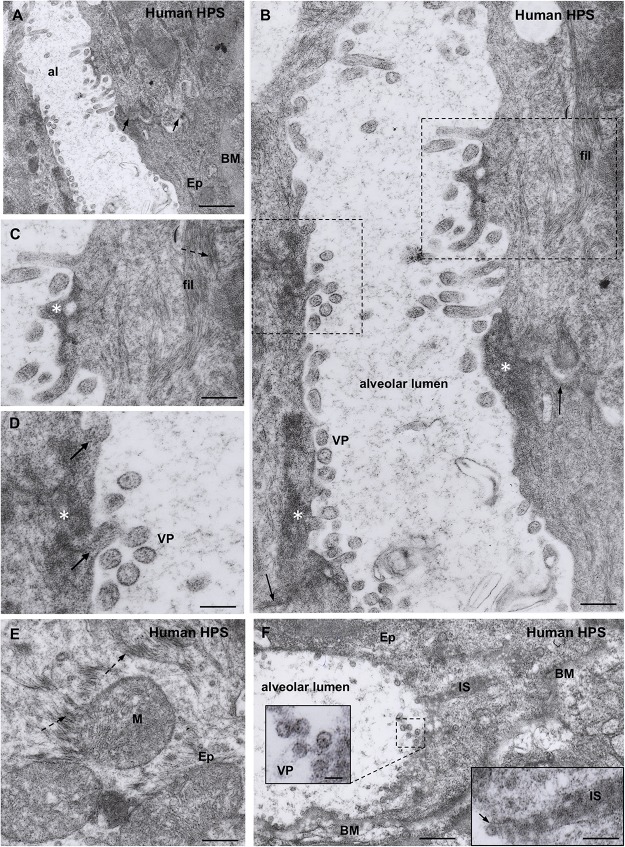
Transmission electron microscopy of human lung from a lethal HPS case. **(A)** Low magnification view of the walls of an alveolum (al). The arrows point to the plasma membrane of neighbor epithelial cells (Ep). BM, basement membrane. Areas of this field are shown at higher magnification in the following figures. **(B)** Alveolar walls of the alveolum shown in **(A)**. The cytoplasm of epithelial cells contains filamentous (fil) and granular (asterisks) inclusions. Virus-like particles (VP) and tubular structures appear lined along the apical domain of the plasma membrane. The arrows point to the plasma membrane of neighbor epithelial cells. **(C)** Detailed magnification of area framed by rectangle in B showing filamentous (fil, arrow) and granular (asterisk) inclusions. **(D)** Detailed magnification of area framed by square in B showing virus-like particles (VP) and granular inclusions (asterisk) that are in continuity with the content of tubular formations projecting to alveolar lumen (arrows). **(E)** Paranuclear region of an epithelial cell (Ep) showing the fine structure of the filamentous inclusions (broken arrows). **(F)** Alveolar epithelial cells (Ep) with isolated or clustered virus-like particles associated to the luminal cell surface. BM, basement membrane; IS, intercellular space filled with densely packed granulo-filamentous material. *Left inset*. Detailed view of virus-like particles (VP). *Right inset.* High magnification of electron dense material occupying a dilated intercellular space (IS) with a virus-like particle located at the luminal end (arrow). *Scale bars.* **(A)** 460 nm; **(B)** 175 nm; **(C)** 140 nm; **(D)** 105 nm; **(E)** 200 nm; **(F)** 370 nm, left inset 75 nm, right inset 180 nm.

**FIGURE 5:**
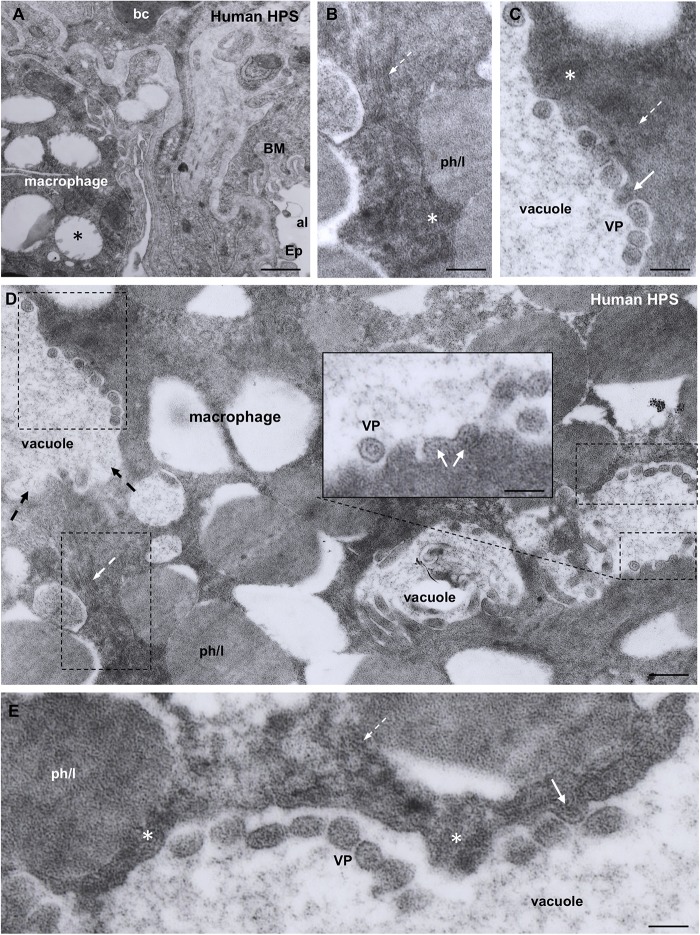
Transmission electron microscopy of human lung from a lethal HPS case. **(A)** Low magnification of an area including a portion of an alveolar epithelial cells (Ep) with its basement membrane (BM), a blood capillary (bc) and an interstitial macrophage displaying large vacuoles (asterisk). **(B)** Detailed view of the area framed by bottom rectangle in D. Filamentous inclusions (broken arrow) occupying the cytoplasm between large phago-lysosomes (ph/l) are seen. **(C)** Area shown in top rectangle of **(D)** showing granulous inclusions near the wall of a vacuole and virus-like particles (VP) protruding into the vacuole lumen (arrow). **(D)** Large area of cytoplasm of a macrophage displaying numerous phago-lysosomes (ph/l) and large vacuoles with virus-like particles inside. *Inset.* Detailed view of a vacuole wall showing virus-like particles (VP) protruding into the lumen or lying free. **(E)** Area shown in right rectangle of **(D)** showing filamentous (broken arrow) and granulous (asterisks) inclusions. Virus-like particles protrude into the vacuole lumen (full arrow) or lie free inside the vacuole (VP). *Scale bars.* **(A)** 2 μm; **(B)** 130 nm; **(C)** 120 nm; **(D)** 230 nm, inset 90 nm; **(E)** 80 nm.

Although essentially the same observations were made in the two cases studied, the following description is based on the fatal case whose lung was fixed in aldehydes shortly after death. Cytoplasmic inclusions and virus particles were seen in the epithelial cells of the alveolar walls and in microphages. Since ultrastructural immunocytochemistry was not performed, the virus particles will be referred to as virus-like particles. Characteristic hantaviral inclusions reported for SNV and other hanta viruses (Tao et al., 1987; Goldsmith et al., 1995; Zaki et al., 1995) were found in the alveolar epithelial cells. These inclusions were: (1) Elongated masses, about 600 nm long and 300 nm width, were formed by densely packed electron dense particles of about 8 nm; these masses localized preferentially close to the luminal domain of the plasma membrane or close to the nucleus (Figures 4B–D). (2) Clusters of filaments, many of which were grouped as pairs; along each pair there were regions of higher electron density, resembling a sort of adherent junction (Figures 4C,E). The granulous and the filamentous masses were spatially closed or mixed, forming elongated granulo-filamentous masses about 2 μm long (Figures 4D, 5B,C,E). Masses of granulo-filamentous material were occasionally seen in dilated zones of the intercellular space of the alveolar epithelium (Figure 4F).

A distinct feature was the presence in the alveolar lumen of virus-like particles, 60–70 nm in diameter, containing fine electron dense material; the virus-like particles lined along the apical domain of the plasma membrane or formed small clusters (Figures 4A–D,F). Short tubular formations, about 60 nm thick, projected to the alveolar lumen; they contain a material similar to the granular inclusions and to the content of the virus particles (Figures 4B,C,D).

Macrophages contained phago-lysosomes and large vacuoles, 1–3 μm in size (Figures 5A,D). The masses of electron dense particles and the bundles of paired filaments, similar to those found in the alveolar epithelial cells, were present in the cytoplasm of macrophages, spatially associated to the large vacuoles (Figures 5B–E). Short tubular formations, about 60 nm thick, projected to the vacuole lumen. Virus-like particles, 60–70 nm in diameter, were in the vacuole lumen, close to the vacuole membrane (Figures 5C–E).

### Hantavirus Antigens Distribution in Submandibular Salivary Glands of ANDV Infected *Oligoryzomys longicaudatus*

**FIGURE 6:**
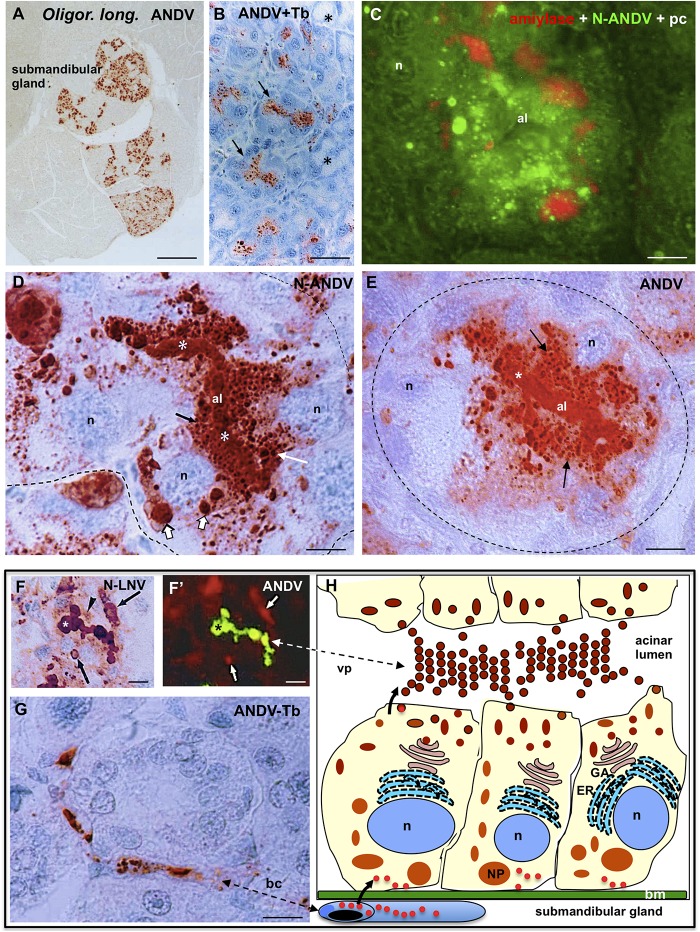
Submandibular salivary gland from infected *Oligoryzomys longicaudatus.* **(A)** Fixation by vascular perfusion with Bouin fluid. Paraffin section immunostained for ANDV antigens. There are lobules strongly immunoreactive while others are completely devoid of virus antigens. X125. **(B)** Section adjacent to previous one immunostained for ANDV and background stained with Toluidine blue. The supranuclear region of cells forming serous acini contain viral granules (arrows). Mucous acini are devoid of virus antigens (asterisks). **(C)** Paraffin section of a serous acinus. Triple channel for phase contrast (pc) and double immunofluorescence for amylase (red) and N-ANDV (green), Acinar cells contain amylase at the base and N-ANDV granules and masses in the supranuclear cytoplasm. n, cell nucleus; al, acinus lumen. **(D)** Section of a serous acinus immunostained for N-ANDV. Virus material is seen as large masses located at base of the cells (large white arrows), as supranuclear granules (white arrow), as fine apical granules (black arrow) and filling the acinus lumen (al, asterisks). n, cell nucleus. **(E)** Section of a serous acinus immunostained for ANDV. ANDV + material appears as numerous supranuclear granules (arrows) and filling the lumen of acinus (al, asterisks). The large masses at the cell base are not visualized. **(F,F’)** Sequential immunostaining of the same section of submaxillary gland from infected *Oligoryzomys longicaudatus*, using anti-ANDV labeled with FITC in the first sequence **(F’)** and anti-N-LNV in the second sequence revealed by the PAP method **(F)**. Material located in the lumen of acinus reacts with both antibodies (asterisks). Anti-N-LNV reacts, in addition, with large intracellular masses located at the cell base (arrows) and granules located near the acinus lumen (arrowhead). **(G)** Blood capillary containing virus antigens. **(H)** Line drawing of a serous acinus of submandibular gland representing some of the findings shown in previous figures. Bm, basement membrane, GA, Golgi apparatus; ER, endoplasmic reticulum; n, cell nucleus; NP, nucleocapsid protein; vp, virus particles. The bent arrows indicate the probable passageway of virus from endothelium to secretory cells and to the acinar lumen. *Scale bars.* **(A)** 400 μm; **(B)** 20 μm; **(C)** 6 μm; **(D)** 5 μm; **(E)** 6 μm; **(F,F’)** 10 μm; **(G)** 8 μm.

Viral antigens were detected in secretory cells of serous acini of the submandibular glands (Figures 6B,C). In sections through the whole gland it became evident that lobules whose acini were loaded with virus antigens were intermingled with others completely devoid of virus antigens (Figure 6A). Double immunofluorescence for amylase, enzyme secreted by serous acinar cells, and N-ANDV showed that the enzyme and the virus material coexisted in the same cells (Figure 6C).

**FIGURE 7:**
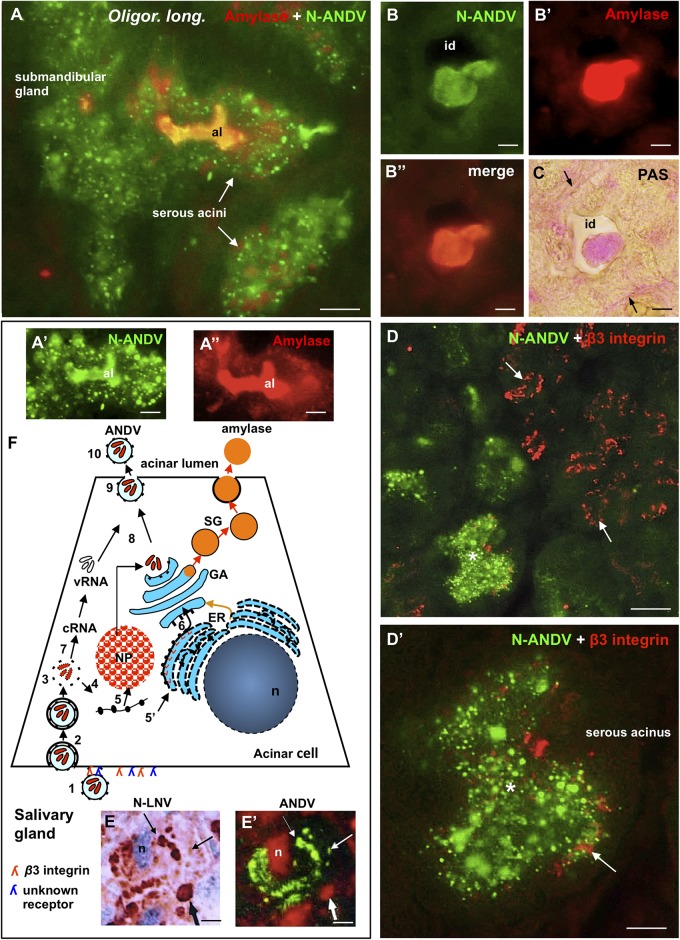
Submandibular salivary gland from infected *Oligoryzomys longicaudatus.* Fixation by vascular perfusion with Bouin fluid. **(A)** Section of serous acini processed for double immunofluorescence for amylase (red) and N-ANDV (green). Acinar lumen (al) is filled with a material reacting with both antibodies (orange). **(A’,A”)** Same acinus as in **(A)**, visualized separately for the N-ANDV and the amylase channels. al, acinus lumen. **(B–B”)** Cross section of an intercalated duct containing a material reacting with antibodies against N-ANDV and amylase. **(C)** Adjacent section to that of **(B)**, processed for the PAS reaction for glycoproteins. The content of the intercalated duct (id) and its basement membrane (arrows) are PAS+. **(D)** Double immunofluorescence of submandibular gland for N-ANDV (green) and β3 integrin (red). There are β3 integrin + acini devoid of virus antigens (arrows) and others that are β3 integrin+, N-ANDV + (asterisk). **(D’)** Detailed magnification of acinus shown in **(D)** (asterisk). β3 integrin + material is at the cell base (arrow) and N-ANDV + masses and granules occupy most of the cytoplasm of serous cells. **(E,E’)** Sequential immunostaining of the same section of a serous acinar cell using anti-ANDV labeled with FITC in the first sequence **(E’)** and anti-N-LNV in the second sequence revealed by the PAP method **(E)**. Material located in the perinuclear region reacts with both antibodies (thin arrows). Large masses located at the cell base (thick arrows) only react with anti-N-LNV (thick arrows). **(F)** Schematic representation of the events that would occur in serous secreting cells of the submandibular gland of *Oligoryzomys longicaudatus* infected with ANDV. Key events of the Hantavirus life cycle largely substantiated in previous investigations (Jonsson et al., 2010; Muyangwa et al., 2015) have been included for the better understanding of the present immunocytochemical findings. *Left part of the chart:* **(1)** Attachment of Hantavirus to the basal domain of plasma membrane of acinar cell through interaction with receptors (β3 integrin; and a second receptor of unknown nature?). **(2)** Entry by endocytosis. **(3)** Uncoating. **(4)** Transcription of viral RNAs. **(5)** Translation of virus cRNA into nucleocapsid proteins (NP) using free ribosomes **(5)** and into virus glycoproteins using membrane-bound ribosomes **(5’)** at endoplasmic reticulum (ER). **(6)** Transport to the Golgi apparatus (GA). **(7)** Replication of virus RNA. **(8, 9)** Assembly of virus components on route to the plasma membrane. **(10)** Exit. *Right part of the chart*: biosynthesis of amylase (53 kDa glycoprotein) at the endoplasmic reticulum (ER), glycosylation and packaging at the Golgi apparatus (orange arrow). Secretory granules (SG) move to the apical plasma membrane and underdo exocytosis. *Scale bars.* **(A)** 7 μm; **(A’,A”)** 5 μm; **(B,B’,B”,C)** 3.5 μm; **(D)** 20 μm; **(D’)** 8 μm; **(E,E’)** 7 μm.

In sections of serous acinus immunostained for N-ANDV, virus antigens appeared as (1) as large immunoreactive masses, 2 a 3 μm in diameter, located at the basal region of the secretory cells; (2) as granules of 0.4–1 μm filling the supranuclear region of the cells; and (3) as a homogeneous mass filling the acinar lumen (Figure 6D). In adjacent sections anti-ANDV reacted with the supranuclear antigen particles and the content of the acinus lumen, but it did not react with the large basal masses (compare Figure 6E). This finding was confirmed by using a different immunostaining protocol. The same section was processed for sequential immunostaining. Anti-ANDV labeled with FITC was used first in the sequence and unlabeled anti-N-ANDV or anti-N-LNV were used second in the sequence and then revealed by the PAP method. The viral material located in the apical granules of the serous secreting cells and that filling the lumen of acini, reacted with both antibodies (Figures 6F,F”). The large basal masses reacted with anti-N-LNV and anti-N-ANDV but did not react with anti-ANDV (Figures 6F,F’, 7E,E’).

Endothelial cells of the blood capillaries contained viral antigens (Figure 6G). The line drawing of Figure 6H summarizes some of the findings described above.

Section of serous acini processed for double immunofluorescence for amylase and N-ANDV revealed that amylase granules and viral material although coexisting in the same cell do no coexist in the same subcellular compartment; however, the lumen of the acinus was filled by both, amylase and virus antigens (Figures 7A,A’,A”). Similarly, the lumen of the intercalated ducts was filled with an amylase +, N-ANDV + and PAS + material (Figures 7B,B’,B”,C).

Double immunofluorescence for N-ANDV and β3 integrin showed that there were β3 integrin + acini devoid a virus material, and β3 integrin + acini loaded with virus antigens (Figure 7D). In the latter, β3 integrin + reaction was at the base of the serous acinar cells (Figure 7D’).

Figure 7F is a schematic representation of the events that would occur in serous secreting cells of the submandibular gland of *Oligoryzomys longicaudatus* infected with ANDV. Key events of the Hantavirus life cycle largely substantiated in previous investigations (Jonsson et al., 2010; Muyangwa et al., 2015) have been included in the drawing for a better understanding and interpretation of the present immunocytochemical findings.

### Hantavirus Antigens Distribution in Submandibular Salivary Glands of HSP Cases

**FIGURE 8:**
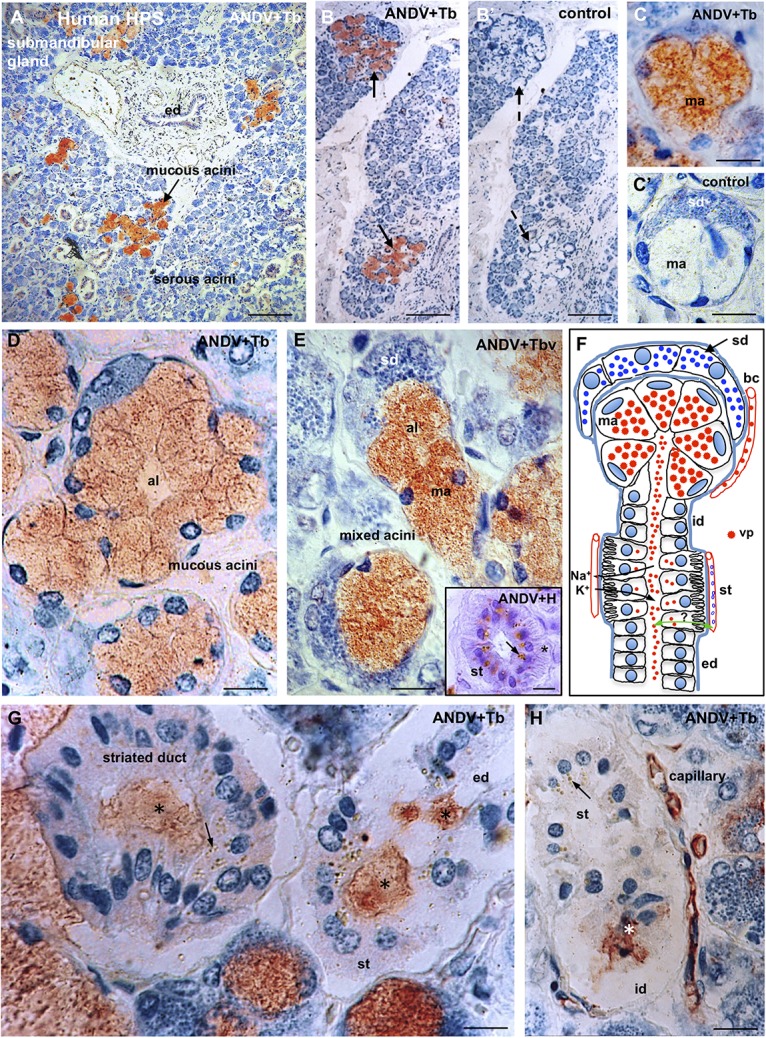
Submandibular salivary gland from lethal Hantavirus pulmonary syndrome cases. **(A–H)** Paraffin sections immunostained with anti-ANDV and background stained with Toluidine blue. **(A)** Low magnification showing that only mucous secreting cells contain immunoreactive hantavirus antigens. **(B,B’)** Adjacent sections processes for the complete immunocytochemical procedure (**B**) and a procedure in which incubation in the primary antibody has been omitted **(B’)**. **(C,C’)** Adjacent sections of a mixed acinus processed for the complete **(C)** and the incomplete (control, **C’**) immunocytochemical procedure. ma, mucous acinus; sd, serous demilune. **(D)** The cells of mucous acini are filled with Hantavirus antigens. Al, acinus lumen. **(E)** Mixed acini. Al, acinus lumen; ma, mucous acinus; se, serous demilune. *Insert*. Cross section of a striated duct immunostained for ANDV and background stained whit hemathoxylin. Viral granules are seen in the supranuclear cytoplasm; at the cell base striations are recognizable (asterisk). **(F)** Line drawing of a mixed acinus and its excretory pathways of human submandibular gland. Virus granules (vp) localize in endothelium of capillaries (bc), mucous cells (ma) and in the lumen of the different segments of excretory pathway, namely, intercalated duct (id), striated duct (st), excretory duct (ed). Cells of striated ducts have numerous infoldings of the basal plasma membrane and numerous mitochondria. At this site, the primary secretion produced in the acinus is modified by inflow of K^+^ and the outflow of Na^+^. Green double-head arrow transcytosis of virus? **(G,H)** Viral antigens are in the lumen (asterisks) of intercalated ducts (id), striated ducts (st) and excretory ducts (ed), and in the cytoplasm of striated cells (arrows) and endothelial cells of capillaries. X1250. *Scale bars.* **(A)** 200 μm; **(B,B’)** 200 μm; **(C,C’)** 15 μm; **(D)** 15 μm; **(E)** 15 μm; inset 15 μm; **(G)** 12 μm; **(H)** 20 μm.

Sections of submandibular glands stained with toluidine blue showed a strong staining of the serous acini; this basic dye has affinity for the ribonucleic acid present in the highly developed rough endoplasmic reticulum of these cells (Figures 8A,B’). Submandibular gland is a mixed gland with serous acini outnumbering the mucous acini. The mucous component of the gland is formed by mucous acini and mucous acini bearing a demilune of serous secreting cells (Figures 8D,E). Sections stained with toluidine blue and immunostained with anti-ANDV clearly showed that hantavirus antigens were confined to the mucous secreting cells (Figures 8A–E).

In the mucous secreting cells, hantavirus antigens appeared as a homogeneous population of fine granules about 0.7 μm in size that distributed evenly throughout the cytoplasm (Figures 8C–E).

The excretory pathway includes the acinus lumen, intercalated, striated and excretory ducts (Figure 8F). Hantavirus antigens were present in the lumen of the whole excretory pathway (Figures 8F–H).

Cells of the striated ducts have numerous infoldings of the basal plasma membrane, what gives this segment of the excretory pathway a striated appearance (Figure 8E, inset). At this site, the primary secretion produced in the acinus is modified by bidirectional transport of ions from and to local blood capillaries (Figure 8F). Striated ducts were the only portion of the excretory pathway whose cells contained hantavirus antigens. The virus granules were located at the supranuclear region of the striated cells (Figures 8E, inset, G,H).

The endothelium of blood capillaries located in the vicinity of the mucous acini and striated ducts contain ANDV + antigens (Figures 8F,H).

Sections in which incubation with the primary antibody had been omitted did not show any immunostaining (Figures 8B,B’,C,C’).

## Discussion

The aim of the present investigation was to make a detailed analysis of the cellular and subcellular distribution of ANDV antigens in the lung and salivary glands of infected reservoir rodents and humans. For this purpose, a set of antibodies raised against ANDV antigens were used in parallel with antibodies against marker proteins. We are aware that detection of ANDV antigens in particular cell types does not necessarily indicate viral infection or replication. The possibility that the material revealed by anti-ANDV antibodies does not correspond to ANDV virions but to phagocytosis has to be considered. Nevertheless, the pattern of immunoreaction produced by the set of anti-ANDV antibodies in individual cells of the lung and salivary glands and the ultrastructural evidence obtained in the lung of HPS cases suggest infection rather than phagocytosis (see below). Furthermore, the presence of ANDV RNA in saliva of infected *Oligoryzomys longicaudatus*, the horizontal transmission in this reservoir rodent, and the person-to-person transmission of ANDV support that the ANDV-immunoreactive material in cells of the salivary glands correspond to viral infection (Padula et al., 2004). Although ultrastructural immunocytochemistry for ANDV was not performed, the following observations suggest that the virus-like particles seen under the electron microscope would correspond to ANDV particles. (1) The ultrastructural features (size, shape, inner structure) of the virus-like particles found in the alveolar lumen close to the apical plasma membrane of cells lining the alveoli are similar to those of other hantaviruses. (2) The alveolar epithelial cells and lung macrophages contain intracytoplasmic granulo-filamentous inclusions regarded as characteristic of hantaviral inclusions by several authors (Tao et al., 1987; Goldsmith et al., 1995; Zaki et al., 1995). (3) The same cell types (alveolar epithelial cells and lung macrophages) of neighboring lung samples processed for light microscopy immunocytochemistry contain ANDV-immunoreactive antigens.

### Subcellular Distribution of ANDV Antigens

Fixation of the lungs and salivary glands of infected *Oligoryzomys longicaudatus* by vascular perfusion resulted in a well-preserved cytology and histology, allowing a fine microscopic analysis. In the serous secreting cells of the submandibular gland, a typical polarized secretory cell type, antibodies against the ANDV nucleoprotein (anti-N-ANDV) reacted with (i) large masses, 2 a 3 μm in diameter, located at the basal cell region, close to the nucleus (LI, large inclusions), (ii) 0.4–1 μm granules filling the supranuclear region, and (iii) a homogeneous material filling the acinar lumen. At variance, the antiserum against cell cultured ANDV (anti-ANDV) reacted with the supranuclear granules and the content of acinar lumen but not with the large masses. The possibility that LI correspond to cytoplasmic inclusions of N protein may be suggested on the following grounds. (1) Four h after infection with hantavirus, N protein starts to be expressed throughout the cytoplasm; 24 h post infection, N protein accumulates in the perinuclear region; by the fifth post infection day, N protein appears as a highly condensed structures localized in the perinuclear region, close to the Golgi-endoplasmic reticulum interphase (Ramanathan et al., 2007). (2) The use of a rabbit polyclonal antibody and a mouse monoclonal antibody against N protein allowed to distinguish between N protein accumulated in the cytoplasm from that associated with the Golgi (Wang et al., 2008). (3) The LI described in the present investigation reacted with the antibody specific for N protein; their lack of reaction with anti-ANDV could be due to low titers of anti-N protein antibodies present in the antisera when a whole preparation of ANDV is used as immunogen. An alternative explanation is that the anti-N protein antibodies present in the anti-ANDV antisera do not react with the N protein present in the cytosol but do react with the N protein packed in the Golgi cisternae or into the virus particles. Interestingly, Wang et al. (2008) concluded that the conformation or molecular interaction of the N protein is different when it is in the cytoplasm or in the Golgi, the site of viral assembly. (4) LI are localized at the basal region of the salivary infected cells, close to the nucleus-rough endoplasmic reticulum, and to the basal domain of the plasma membrane where the entry of virus would occur (see receptor session). (5) LI are missing from the apical cytoplasm, where small antigen granules accumulate. These observations would suggest that salivary cells bearing LI, in addition to the virus antigen particles distributed throughout the cytoplasm, are cells undergoing viral infection and replication (Figure 7F).

Granular, filamentous and granulo-filamentous cytoplasmic inclusions, as seen with transmission electron microscopy and confirmed by immunocytochemistry, are regarded as a common morphological marker for 13 strains of hantaviruses studied, irrespective of their origin (Tao et al., 1987). The presence of these cytoplasmic inclusions is regarded as evidence of viral replication. In HPS patients infected with SNV, the viral granulo-filamentous inclusion were found in the endothelial cells of the lung microvasculature (Zaki et al., 1995); these authors indicated that this was consistent with SNV replication in the endothelial cells. The transmission electron microscopic study of the lung of a fatal case of HPS caused by ANDV (present investigation) revealed the presence in the alveolar epithelial cells of granular, filamentous and granulo-filamentous cytoplasmic inclusions similar to those reported in cells infected with hanta viruses different from ANDV (see above). This, and the presence in these cells of ANDV-immunoreactive small and large cytoplasmic inclusions support that ANDV infection and replication would take place in the alveolar epithelium.

The ultrastructure of the lung macrophages of patients with HPS caused by ANDV revealed granulo-filamentous cytoplasmic inclusions similar to those seen in the alveolar epithelium; this and the immnunoreactivity of these cells with ANDV antibodies suggest that macrophages are also a site of ANDV infection and replication. Typical 60 nm virus particles were seen protruding into the lumen of large vacuoles of macrophages. A similar observation was reported by Zaki et al. (1995). The fate of these virus-like particles is difficult to envisage. Would they finally be secreted or degraded? Considering that alveolar macrophages carrying viral particles are migratory cells that can reach large segments of the airways, their expectoration becomes a deadly bullet for ANDV transmission (see person-to-person transmission section).

### Entry of Hanta Virus Into Host Cells and Receptors

Hantaviruses infect endothelial, epithelial, macrophage and follicular dendritic cells through the interaction of the viral glycoprotein with the receptor(s) of the host cell (Jonsson et al., 2010). Strong evidence points to integrins as the receptor for hantaviral glycoproteins. However, there are findings indicating that other receptors may also be involved in virus entry.

*In vitro* and *in vivo* evidence indicates that β3 integrin facilitates the cellular entry hantaviruses associated with HPS (Gavrilovskaya et al., 1998; Song et al., 2005). However, integrins may not be the exclusive receptors for hantavirus infection, since cells without β3 integrin proteins permit infection (Mou et al., 2006; Jonsson et al., 2010; Ray et al., 2010). Furthermore, murine β3 integrin does not serve as a receptor for pathogenic hantaviruses (Raymond et al., 2005). Since hantaviruses can replicate in mice, virus receptors other than β3 integrin exist. Membrane proteins of the complement regulatory system, such as the decay-accelerating factor (Krautkrämer and Zeier, 2008; Ermonval et al., 2016), a novel 70 kDa protein (Mou et al., 2006), an unknown 30 kDa protein (Kim et al., 2002) have been suggested as alternative receptors or co-receptors for hantaviruses.

The present findings in the submandibular salivary gland of *Oligoryzomys longicaudatus* infected with ANDV showed that most, but not all acini display immunoreactive β3 integrin at the basal plasma membrane domain of the serous secreting cells. Surprisingly, about half of the β3 integrin + acini contained virus antigens while the others were antigen-free, suggesting that β3 integrin alone does not allow ANDV entry, and that other receptors or co-receptors would be required (Figure 7F). In human salivary glands, mucous and serous acinar cells display immunoreactive β1 and β3 integrin at the basal cell pole (Loducca et al., 2003). However, only mucous acini of the human submandibular gland are infected by ANDV (present report), indicating that also in humans β3 integrin alone does not allow ANDV entry.

### There Are Significant Differences in the Cellular Distribution of SNV and ANDV in Their Respective Rodent Reservoirs

Using an antiserum against SNV nucleocapsid protein, Green et al. (1998) investigated immunohistochemically the localization of virus antigen in tissues of infected deer mice (*Peromyscus maniculatus*), the natural reservoir of SNV. Virus antigens were only detected in endothelial cells of heart and lung. However, other studies showed a wide tissue distribution of SNV in deer mice naturally (Botten et al., 2002) or experimentally (Botten et al., 2000) infected with SNV. Both, viral antigen and viral RNA were detected in the lung, heart, kidney, salivary glands and other organs. In all these tissues, the cells containing the viral antigen had the morphological characteristics of endothelial cells (Botten et al., 2000). Unfortunately, neither the nature of the salivary gland infected nor the cell types hosting the virus in this gland are mentioned in these studies.

In publications dealing with the SNV distribution in cells and tissues of infected deer mice, no mention is made about macrophages or epithelial cells, especially those of the alveolar epithelium of the lung. Thus, it might be inferred that SNV was not detected in these cells. This a key difference with ANDV cell tropism. In *Oligoryzomys longicaudatus* rodents infected in nature, ANDV antigen also localizes in endothelial cells of several organs, namely, heart, kidney, brain, brown adipose tissue (data not shown), lung and submandibular salivary gland. But, the bulk of ANDV antigen localizes in epithelial cells of lung alveoli and salivary gland, and in lung macrophages (present report). This supports the view that in *Oligoryzomys longicaudatus*, ANDV may be principally transmitted via pulmonary droplets and saliva (Padula et al., 2004). The differential distribution of SNV and ANDV in the lung of their respective rodent reservoir may explain the important differences in the mechanism of rodent-to-rodent virus transmission. Of 54 attempts to transmit infection by cohousing *Peromyscus maniculatus* infected with SNV with seronegative cage mates, only one case of transmission occurred (Botten et al., 2002). At variance, ANDV transmitted efficiently among cage mates *Oligoryzomys longicaudatus* (Padula et al., 2004). Furthermore, transmission of SNV occurs primarily via a horizontal mechanism by wounds among deer mice, while in *Oligoryzomys longicaudatus* wounds do not play a major role but other factors, such as grooming, or aerosol spread do (Botten et al., 2002; Padula et al., 2004).

### SNV and ANDV, Both Causing Hanta Pulmonary Syndrome, Have Key Differences in Their Cellular Distribution in the Lung and Salivary Glands of HPS Cases

In a compressive study of SNV distribution in several organs and tissues of HPS cases, Zaki et al. (1995) consistently found hantavirus antigens to be present in capillary endothelial cells in various tissues throughout the body. The endothelium of pulmonary capillaries showed the strongest viral immunoreactivity (Zaki et al., 1995; Green et al., 1998; Toro et al., 1998). Viral antigen was also found in macrophages of the lung interstitium (Zaki et al., 1995; Toro et al., 1998). The presence of SNV antigen in the alveolar epithelium was not reported in these publications; no mention was made about salivary glands. Hanta viral RNA was detected by a sensitive *in situ* PCR method in endothelial cells and pneumocytes of three fatal cases of HPS of New York (Nuovo et al., 1996). To our knowledge, this is the only publication reporting the presence of hantavirus in pneumocytes of fatal cases of HPS.

The present investigation reveals relevant differences in the cell distribution of ANDV in HPS cases as compared with that of SNV in HPS cases. Although ANDV antigens also localizes in capillary endothelial cells of the lung and other organs, the strongest ANDV immunoreactivity is found in the alveolar epithelium (pneumocytes), in macrophages of the alveolar septa and alveolar lumen, and in mucous secreting cells of the submandibular gland. The electron microscopic analysis confirmed that the hantaviral inclusions and virus-like particles are present in the alveolar epithelium of patients infected with ANDV. This key difference between SNV and ANDV could help to explain that only ANDV is transmitted from person to person (see below).

### ANDV Is Exceptionally Transmitted From Person to Person

Until 1996, person-to-person transmission had not been reported for any hantaviruses. In an outbreak of 20 cases that occurred in Southern Argentine, the epidemiologic evidence strongly suggests person-to person-transmission of ANDV (Wells et al., 1997). Soon, direct genetic evidence for ANDV person-to person-transmission was obtained (Padula et al., 1998). This way of transmission was confirmed in outbreaks occurring in Southern Chile (Martínez et al., 2005; Ferrés et al., 2007). Although ANDV clearly is transmitted directly from human to human, how exactly this occurs, or why other pathogenic hantaviruses are not transmitted between humans, is not fully understood.

Considering that ANDV incubation period is about 15–24 days; that during this period there are no clinical signs of disease; and that a person infected by another person may develop the severe phase of HPS shortly after the infecting person did, indicate that that person-to-person spread of the virus takes place during the prodromal phase. Indeed, epidemiologic and genetic data have shown that close and prolonged contact occurred during the prodromal phase (Martínez et al., 2005). Furthermore, person-to- person transmission requires prolonged, intimate interpersonal contact (Wells et al., 1997; Martínez et al., 2005; Ferrés et al., 2007).

How does ANDV actually spreads between persons? The presence of the virus in pneumocytes and macrophages of the airway, and in the salivary glands suggest that ANDV spread through saliva or coughing, as discussed below.

### In Patients With HPS Caused by ANDV the Alveolar Epithelium and Macrophages Could Be the Gate for the Airway Spreading of the Virus

The early interactions of hantaviruses within the respiratory tract are poorly understood. Following inhalation, the first barrier that the virus meets is the mucus layer covering epithelial cells of the respiratory airway. Then the virus encounters a second barrier, the alveolar epithelium (Pettersson, 2015). It is not clear how the virus traverses the respiratory epithelium to initiate infection in the endothelium (Rowe and Pekosz, 2006).

In ANDV infected patients the virus is present in pneumocytes, with an intracellular pattern of distribution of immunoreactive antigens and the presence of cytoplasmic inclusions and virus-like particles that suggests viral infection and replication (Figure 3D). What is the source of the virus present in pneumocytes? What is the fate of virus present (and probably replicating) in pneumocytes? The findings of Rowe and Pekosz (2006) may help to understand these open questions. These authors used infection of primary, differentiated airway epithelial cells of hamster to investigate the capacity of ANDV to interact with and cross the respiratory epithelium. They found that ANDV successfully infected both the apical and the basolateral side of polarized epithelial cells, with bidirectional secretion of virus, even though apical infection and release were favored. Rowe and Pekosz (2006) concluded that the respiratory epithelium could serve as a site of ANDV entry and replication, and that a bidirectional virus secretion follows, allowing the virus to have access to endothelium and exit through the airway. Apical infection and basolateral release of viruses were observed when an *in vitro* model of human lung tissue was infected with ANDV (Sundström, 2013). The present ultrastructural study supports that in the lung of ANDV infected patients the virus replicate in the alveolar epithelial cells and the virus particles are discharged into the alveolar lumen.

Two distinct macrophage populations exist in the lung, termed alveolar macrophages and interstitial macrophages. They have a different origin, phenotype and life cycle (Byrne et al., 2015). Alveolar macrophages in the lung of HPS patients are a key component of the pathogenic mechanism. The presence in the lumen of alveoli and bronchi of numerous and large macrophages containing carbon particles and virus antigen indicates that these macrophages have incorporated the virus while residing in the alveolar lumen. For this to occur, the virus must be present in the alveolar lumen (Figure 3D; Toro et al., 1998). The virus may have reached the lumen of alveoli by secretion from pneumocytes, as supported by the present immunocytochemical and ultrastructural study, or through the airway after inhalation. The latter possibility is supported by the long life of alveolar macrophages, in man being about 80 days (du Bois, 1985) and in mice being similar to life span of the animal itself (Murphy et al., 2008). It may be suggested that in ANDV-infected persons and in *Oligoryzomys longicaudatus*, most or all of the alveolar macrophages were residing in the alveolar lumen at the time of infection. Once the alveolar macrophages have gained access to the bronchial ciliated epithelium (Figure 3C) they move up the airway, where they can be expectorated (Lehnert, 1992). Considering they these macrophages carry viral particles (see above), their expectoration is a deadly bullet for ANDV transmission.

In brief, the presence of virus-like particles in the alveolar space and ANDV antigens in alveolar-bronchial macrophages strongly supports the person-to-person transmission through the respiratory pathway. This transmission would only require close vicinity, rather than needing a close intimate contact, as the one mediating transmission via saliva. This airway of person-to-person transmission could help to explain the recent outbreak of HPS in southern Argentina. Epuyén is a small village located in the Chubut Province of Patagonia; it has a population of approximately 2,000 persons. A total of 29 laboratory-confirmed cases of HPS, including 11 deaths occurred between October 28th, 2018 and January 20th of 2019. Seven patients attended the same party on November 3rd and started with the symptoms 3 weeks later. Additional 17 cases were epidemiologically linked to these confirmed cases. One of the confirmed cases was a healthcare worker who moved for 1 day from Palena Province (Chile) to Epuyén to care for a later confirmed case while she was in her prodromal phase (source: Published by the Ministry of Health of Chubut Province and reproduced by PAHO/WHO)^3^.

### In Patients With HPS Caused by ANDV the Salivary Glands Appear as a Target for Virus Replication and Exit Pathway Through Saliva

RNA of SNV was detected in the saliva, but not the urine and feces, of a series of experimentally infected and in one naturally infected deer mice (*Peromyscus maniculatus*) (Botten et al., 2002). In these animals, N protein was immunocytochemically detected in various organs, including the salivary glands (Botten et al., 2002). In the natural reservoir of ANDV, the mouse *Oligoryzomys longicaudatus*, viral RNA of ANDV was detected in saliva of seropositive, but not in urine samples (Padula et al., 2004). ANDV antigen was described to be present in the salivary gland (Padula et al., 2004). Neither of these two reports mentioned the type of salivary gland and cell types containing ANDV.

The present investigation has shown that in infected *Oligoryzomys longicaudatus* ANDV selectively infect the submandibular gland, with virus antigen being present in endothelial cells of capillaries, in the serous secreting cells and filling the lumen of the excretory pathway. Thus, the previous and the present findings indicate that saliva is the preferred route of transmission of SNV and ANDV between their natural hosts, *Peromyscus maniculatus* and *Oligoryzomys longicaudatus*, respectively. These findings also indicate that hantaviruses are transmitted from reservoir to human though rodent saliva (Pettersson et al., 2008).

Apart from our early preliminary report on the immunocytochemical detection of ANDV antigen in salivary gland of HPS cases (Navarrete et al., 2007), we have found no other publication dealing with the presence of RNA or antigen of ANDV in salivary glands or saliva of HPS cases. The present findings show that in HPS cases, ANDV antigen is present in capillary endothelium, and selectively localized in the mucus acini of the submandibular gland, with the bulk of immunoreactive antigens in the mucous secreting cells and in the lumen of the excretory pathway (Figure 8F). Interestingly, few antigen granules were consistently present in the supranuclear region of the cells forming the striated duct. At this discrete portion of the duct system, there is a bi-directional flux of ions, between blood capillaries and duct lumen, and transcytosis of compounds such as IgA (Amano et al., 2012). Whether ANDV undergo transcytosis at the striated duct, from or to the duct lumen remains as an interesting open question (Figure 8F).

Puumala virus (PUUV) antigen has been found in endothelial cells of the parotid gland of a case of hemorrhagic fever and renal syndrome (Pettersson et al., 2011), and PUUV RNA has been detected in saliva from PUUV infected patients (Pettersson, 2015). However, no person-to-person transmission appears to occur with PUUV. There is evidence that human saliva inhibits PUUV replication (Pettersson, 2015). Interestingly, ANDV is less sensitive than PUUV to the antiviral effect of human saliva. This, and the evidence that in PUUV-infected patients, the virus antigen was only visible in the endothelial cells of the salivary glands (Pettersson, 2015), might explain why ANDV but not PUUV, is transmitted between humans (Hardestam et al., 2009).

In brief, the convincing evidence that ANDV is transmitted from person to person (see above), and the present evidence that ANDV is likely to be secreted into human saliva, allow proposing that saliva is involved in human-to-human transmission.

## Conclusion

The present investigation provides evidence that human-to-human transmission of ANDV occurs thought both, the respiratory airway and saliva.

## Author’s Note

This article is dedicated to a close friend, Sonia Iriart de Donoso, who became the first fatal case of Hanta Pulmonary Syndrome in Valdivia, before we were aware of Hanta in this part of the world.

## Data Availability Statement

The datasets generated for this study are available on request to the corresponding author.

## Ethics Statement

The studies involving human participants were reviewed and approved by Comité de Ética, Universidad Austral de Chile, Valdivia, Chile in 1999. Written informed consent for participation was not required for this study in accordance with the national legislation and the institutional requirements. The animal study was reviewed and approved by Comité de Bioetica Universidad Austral de Chile, Valdivia, Chile.

## Author Contributions

RM, PP, LZ, and ER conceived the study. ER and CO designed the study. MN, CC, CM, and MT provided the human samples collected in Valdivia and Coyhaique. RM and EP provided the *Oligoryzomys longicaudatus* samples. EP, MN, ER, CM, and PS performed the immunocytochemical study. ER and MN carried out the ultrastructural study. EP, MN, LZ, PP, CO, and ER analyzed and discussed the data. ER, CO, and LZ wrote the manuscript.

## Conflict of Interest

The authors declare that the research was conducted in the absence of any commercial or financial relationships that could be construed as a potential conflict of interest.

## Footnotes

1 https://www.who.int/csr/don/23-January-2019-hantavirus-argentina/en/

2 www.lal.org.uk

3 https://www.who.int/csr/don/23-January-2019-hantavirus-argentina/en/
